# An Innovative Method for Forming Balls by Cross Rolling

**DOI:** 10.3390/ma11101793

**Published:** 2018-09-21

**Authors:** Zbigniew Pater, Janusz Tomczak, Tomasz Bulzak

**Affiliations:** Department of Computer Modelling and Metal Forming Technologies, Faculty of Mechanical Engineering, Lublin University of Technology, Nadbystrzycka 36, 20-618 Lublin, Poland; j.tomczak@pollub.pl (J.T.); t.bulzak@pollub.pl (T.B.)

**Keywords:** cross rolling, steel balls, fem, experiment

## Abstract

The paper describes an innovative cross rolling method that enables the production of six balls at the same time, each ball with a diameter of 100 mm. The principle of the proposed rolling technique is discussed and the tools used in this forming process are described. Two variations of the proposed method for producing balls were investigated, one performed with the use of flat tools and the other with the use of two rolls. Results of the numerical modelling are discussed. They clearly demonstrate that the proposed method can be used to produce balls with large diameters. Rolling experiments were performed under laboratory conditions to produce 40 mm diameter balls, i.e., in the 1:2.5 scale. The experimental findings show a good qualitative agreement with the numerical results.

## 1. Introduction

Steel balls are used as grinding media in ball mills for milling different materials such as metal ores, coal, gravel and worn-out moulding sands. The yearly demand for these parts, which are manufactured in the diameter range between 25 and 125 mm, amounts to hundreds of thousands of tons.

Currently, balls are produced by casting (predominantly with the use of permanent moulds), die forging and rolling. The accuracy of steel balls used for grinding media is not high (diameter tolerance is ±3 mm). According to Stalinskii et al. [1], the best results are obtained with the rolling method. The rolling process is not used for producing high precision balls used in a ball screw feed drive system, rolling bearings, measuring instruments and tools [2,3,4,5]. Balls for precision applications are manufactured by forging and machining or by machining only.

Rolling processes for balls are predominantly performed on skew rolling mills equipped with helical rolls. At least one ball is formed in one revolution of the rolls, which leads to a very high throughput of this forming process, which is practically only limited by the applied forming temperature. The most difficult part in the design of skew rolling processes pertains to the design of tool shape ensuring that the rolling process is run correctly. This design rests on the assumption that the volume of material constrained in the roll pass is constant and equal to the volume of the ball. The basic design of helical rolls was developed relatively long ago and can be found in the specialist literature (e.g., [6,7,8,9,10]). In addition, recent years have witnessed a growing use of numerical modelling to investigate the stresses and strains in balls produced by hot [11] and cold working processes [12], as well as to analyse the force parameters in these processes [13]. At the same time, attention has been paid to the lack of effective modelling methods with respect to separating balls; the reported solutions are simplified and consist in connecting balls with the use of small diameter cylindrical connectors to form “chains”.

Apart from skew rolling, balls can also be produced by cross wedge rolling (CWR). This forming method is less efficient than skew rolling, however it can be realized with the use of commercial rolling mills manufactured by numerous companies. In a standard CWR process, several balls are formed simultaneously by wedge-shaped tools. Balls are formed from cylindrical rods [14]. Given the universality of the cross wedge rolling technique, it was possible to design its variation enabling the production of 70 mm diameter balls from the heads of scrap railway rails. This rolling process is realized in two stages. First, the head of the rail is shaped into a 52 mm diameter rod; after that, the rod is formed into four balls with a diameter of 70 mm [15]. To this end, a reverse flat-wedge rolling mill was designed which can be operated without idling [16].

Commercial wedge rolling mills also enable the manufacturing of balls of larger dimensions, i.e., with a diameter of 100 mm or larger. This can be done with the use of innovative tools that shape the workpiece with their concave working surfaces, which is typical of cross rolling. This paper presents two variations of the proposed rolling method, in which six balls with a diameter of 100 mm are produced. The innovative aspect of the proposed methods for producing balls lies in the fact that they enable the formation of several (even up to several dozen) balls simultaneously, the number of balls depending on the tool width. In addition, the proposed design of tools (rolls or flat segments) is innovative on a global scale. The tools are made of flanges with concave walls (the number of flanges is by one higher than the number of the balls being formed), which, first, form spherical necking between adjacent balls and, then, effectively separate the balls.

## 2. Numerical Analysis

The proposed rolling method for producing six balls with a diameter of 100 mm was modelled numerically using the commercial simulation software package Forge NxT 1.1. In the numerical analysis, balls were formed from 70Mn3 steel according DIN, the material model of which was obtained from material data library of the software. This model was defined by the Hensel–Spittel equation:

(1) $$\sigma_{F}=1602.58e^{-0.00281T}\varepsilon^{-0.11358}e^{-0.05174/\varepsilon }{\dot{\varepsilon }}^{0.15046},$$

where *σ_F_* is the yield stress, MPa; *ε* is the effective strain, -; *T* is the temperature, °C; and $\dot{\varepsilon }$ is the strain rate, s^−1^.

In the analysis, the Tresca friction criterion was used, defined as:

(2) $$\tau =m\frac{\sigma_{i}}{\sqrt{3}}\frac{\Delta v}{\left|\Delta v\right|},$$

where *τ* is the tangent stress on contact surface, MPa; *m* is the friction factor (set equal to *m* = 0.8); *σ_i_* is the effective stress, MPa; and Δ*v* is the slip velocity on contact surface, mm/s.

As regards other parameters of the rolling process, the temperature of the tools was set equal to 250 °C, the heat transfer coefficient between the tools and the material was set equal to 10 kW/m^2^·K and the friction factor was set to 0.8. The billet was a cylindrical rod of 90 mm in diameter and 520 mm in length, preheated to the temperature of 1100 °C. Two variations of the proposed rolling method were investigated: rolling with the use of flat tools and rolling with the use of two rolls.

What presents some difficulty in numerical modelling of the rolling process for balls is the modelling of separating balls. In most commercial simulation software for modelling metal forming processes, the computations do not continue after the separation of material into two or more parts. For this reason, it is common practice [10,11,12,13,17,18] to apply a simplification that consists in modelling chain-like connections between balls with the use of small diameter cylindrical connectors. This limitation does not occur in Forge NxT 1.1 [15], in which ductile fracture is modelled via setting the boundary value of the Cockcroft–Latham integral that is usually determined by tensile or compression testing. Nevertheless, the boundary values of this integral determined in the above tests are underrated, when compared to cross rolling processes that are characterized by a totally different state of stress [19]. Consequently, based on the results in [20], the boundary value of the ductile damage function was set equal to 3.

### 2.1. Rolling Process with the Use of Flat Tools

**Figure 1:**
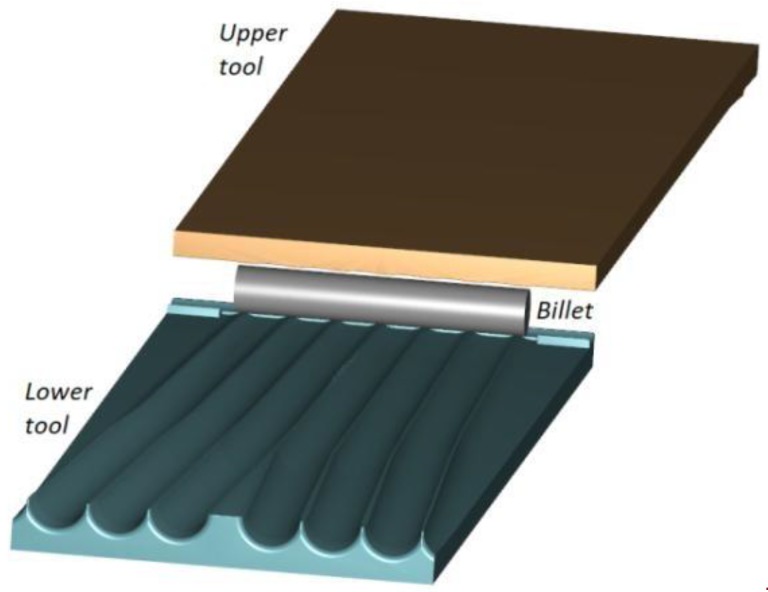
Geometric model of the rolling process for producing six balls of 100 mm in diameter with the use of flat tools.

The rolling process for producing 100 mm diameter balls can be carried out with the use of flat tools of which at least one can perform the reciprocating motion. Figure 1 shows the geometric model of this process, wherein the lower tool is fixed while the upper tool moves at a speed of 0.5 m/s. Apart from the tools, the model consists of a billet modelled by tetragonal finite elements.

**Figure 2:**
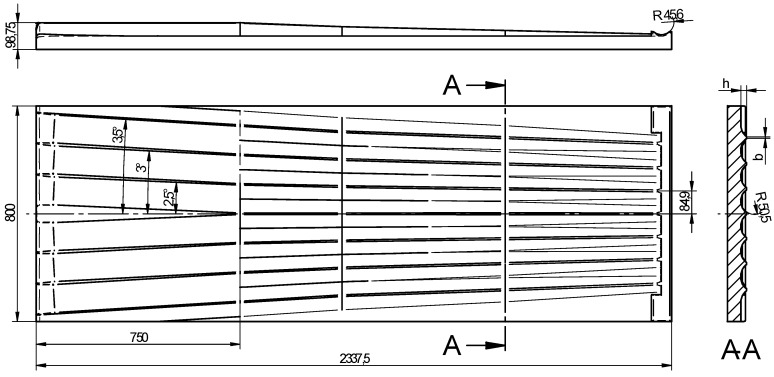
Schematic design of the flat tool for forming six balls of 100 mm in diameter.

The efficiency of the rolling process for balls primarily depends on the shape of the tools; one of these tools is shown in Figure 2. This tool consists of two parts: the forming part (1587.5 mm in length) and the sizing part (750 mm in length). In the forming zone, the workpiece is formed by flanges described by a height *h* and a width *b* as well as concave side walls, the curvature of which is described by the radius of the ball. The dimensions of the flanges, including the spacing between them, are selected such that the volume of material constrained in the roll pass formed by two adjacent flanges is maintained constant. This volume of material is naturally equal to the volume of the ball. The height of the flanges is maintained constant in the sizing zone yet the flanges are deflected from the centre line of the tool (corresponding to the direction of tool motion) by a specified angle (2.5–3.5°) to induce a change in the axis of rotation of the ball. In this region of the tool, any workpiece shape inaccuracies should be removed due to the action of the side walls of the flanges.

Discussing the proposed flat tools for rolling six balls with a diameter of 100 mm, it should be mentioned that, due to their dimensions, these tools can be mounted in rolling mills available on the market. These tools can be mounted in rolling mills manufactured in Belarus [21], e.g., ПМ5.135 (Physical-Technical Institute of the National Academy of Sciences of Belarus, Minsk), WRL10025TS (AMTengineering Ltd. Minsk, Belarus), and SP5000-2-IH (JSC “Beltechnologia & M” Minsk, Belarus), and FBQ100/2500 (Beche & Grohs GmbH - Schuler Pressen GmbH, Remscheid, Germany).

**Figure 3:**
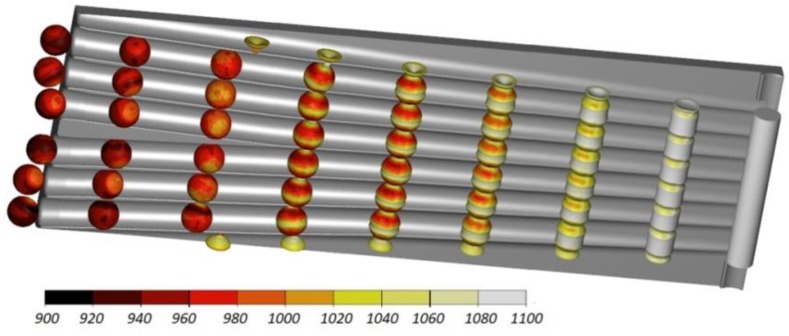
Workpiece shape progression during the cross rolling process for six balls with a diameter of 100 mm, and the distribution of temperature (in °C).

Numerical results demonstrate that the proposed rolling method can be used to produce 100 mm diameter balls for grinding media. Figure 3 shows the rolling process performed in accordance with this method. It can be observed that, due to the action of the flanges, cylindrical neckings are produced, the diameter of which decreases as the process advances. The material extruded from these neckings is moved to the balls and undergoes partial upsetting (the diameter of the ball is 10 mm bigger than that of the billet). At the beginning of the sizing zone, the balls are separated and the scrap material (amounting to approximately 4% of the billet) is cut off. At the end of the process, the balls undergo sizing in the roll passes. The rolling process is stable and there occur no failure modes such as uncontrolled slipping.

**Figure 4:**
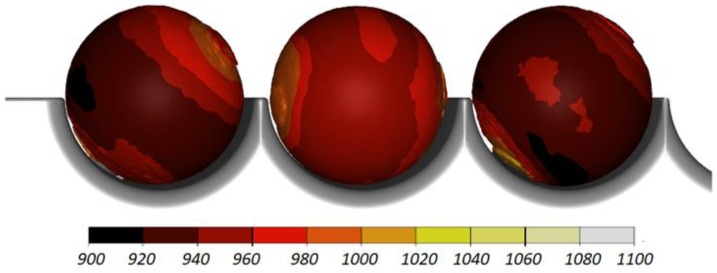
Shape of 100 mm diameter balls produced by cross rolling with flat tools and the distribution of temperature (in °C); the balls are shown in the order from ball in the middle (**left**) to ball at the side (**right**).

Figure 4 shows the shape of balls produced by cross rolling with the flat tools. One can notice small remnants of the material in the place where the balls were separated, which have the shape of small diameter cylindrical protrusions. Equivalent to the remainder of flash in balls produced by forging, these protrusions are acceptable, and the ball size is within the desired manufacturing tolerance of ±3 mm.

**Figure 5:**
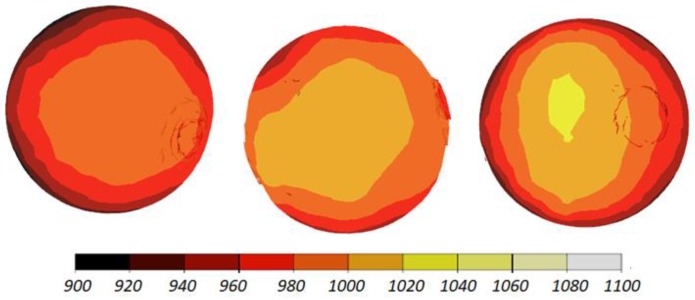
Distribution of the temperature (in °C) inside 100 mm diameter balls produced by cross rolling with flat tools; the balls are shown in the order from ball in the middle (**left**) to ball at the side (**right**).

**Figure 6:**
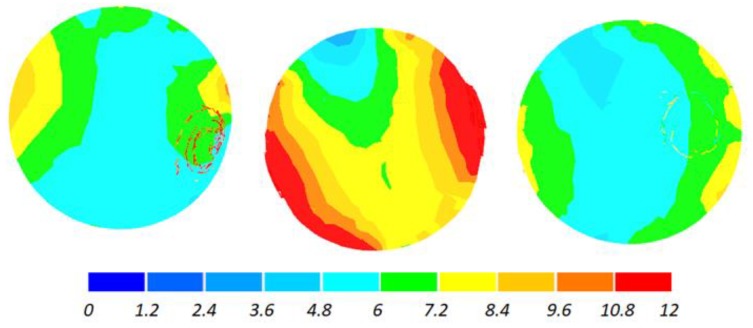
Distribution of effective strains inside 100 mm diameter balls produced by cross rolling with flat tools; the balls are shown in the order from ball in the middle (**left**) to ball at the side (**right**).

**Figure 7:**
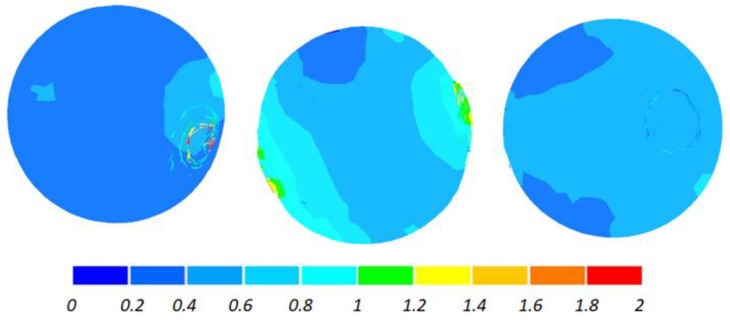
Distribution of the damage function (according to the Cockcroft–Latham ductile damage criterion) inside 100 mm diameter balls produced by cross rolling with flat tools; the balls are shown in the order from ball in the middle (**left**) to ball at the side (**right**).

The data given in Figure 4 and Figure 5 demonstrate that the temperature of the workpiece ranges between 940 and 1040 °C. This means that, prior to quenching, the balls must be kept in free air to equalize and decrease their temperature to approximately 860 °C. Such a high temperature of the workpiece material—despite a relatively long forming time (approximately 9 s)—can be explained by high thermal inertia of produced balls (ball weight is approximately 4.1 kg) and relatively high amounts of heat generated by friction and deformation work. It should however be mentioned that the material inside the balls is not uniformly deformed over its entire volume (Figure 6). The highest strains occur in the regions adjacent to the connectors, while the lowest in the centre of the balls. Apart from that, one can observe significant differences between the distributions of the strains in individual balls. Examining the variations in the damage function shown in Figure 7, it can be observed that the value of this function inside the balls is much smaller than the assumed critical value of 3. Consequently, balls produced by this method should be free from the undesired internal cracking.

**Figure 8:**
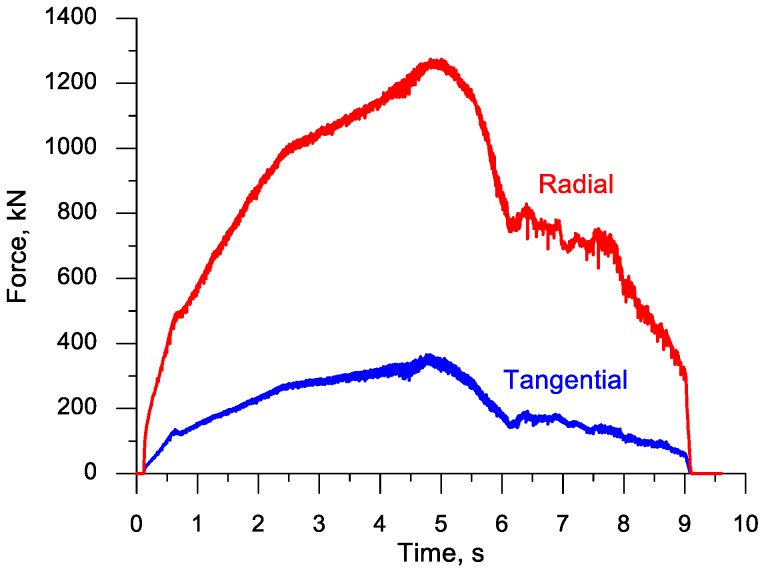
Variations in the radial and tangential forces in the cross rolling process for producing six balls with a diameter of 100 mm.

The plotted data for the forming forces shown in Figure 8 demonstrate that both the radial force (responsible for the accuracy of rolling) and the tangential force (responsible for the motion of the upper tool) are the highest in the fifth second of the process, i.e., in an advanced stage of the forming process. Following the separation of material, the forces rapidly decrease to a significant degree. This decreasing trend continues during the ball sizing stage.

### 2.2. Rolling Process with the Use of Two Rolls

**Figure 9:**
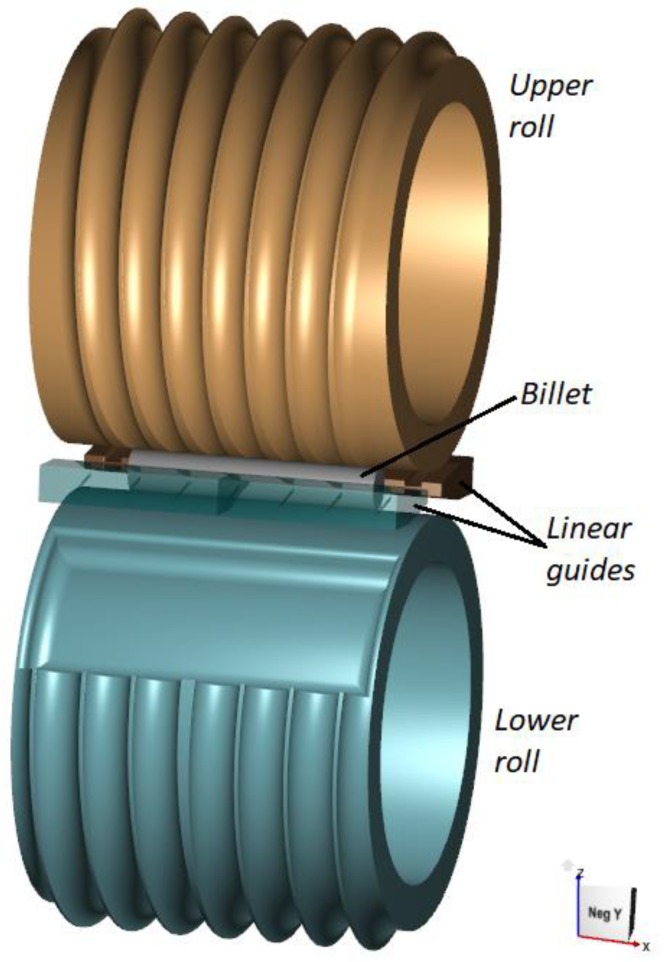
Geometric model of the cross rolling process for producing six balls of 100 mm in diameter with the use of two rolls.

Balls of 100 mm in diameter can also be produced with the use of two rolls rotated in the same direction at the same rotational speed. A geometric model of this rolling process is shown in Figure 9. In contrast to rolling with the use of the flat tools, the rolling process with two rolls requires the use of linear guides to maintain the workpiece in the working space of the forming tools. A considerable advantage of the rolling process based on the use of two rolls is the lack of idling (return motion) of the tools, as was the case in the previously discussed rolling process.

**Figure 10:**
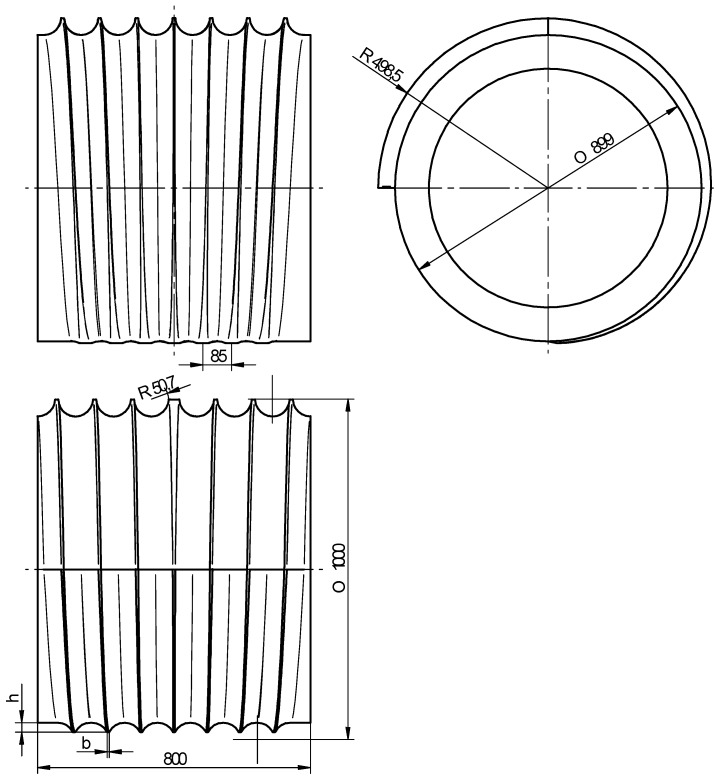
Schematic design of a roll for forming six balls with a diameter of 100 mm.

Figure 10 shows a schematic design of one of the rolls used in the proposed method for producing six balls with a diameter of 100 mm. This tool is also provided with flanges, the height of which increases in the midpoint of the circumference of the rolls (in the so-called forming zone). The flange height is however maintained constant in the sizing zone that is located ¼ of the circumference of the rolls. The remaining part of the circumference of the tools creates the so-called input–output zone, which is indispensable for feeding billet and removing finished balls. With the automated feed of the billet, the rolls designed in this way allow for operation in a continuous mode, with the rotational speed of the tools set equal to 5 rev/min. In this operating mode, the rolling output amounts to 30 balls per minute, i.e., approximately 7.3 tons per hour.

In the proposed solution, the axial spacing between the rolls (when the roll face length is minimum 800 mm) is set equal to 1000 mm. There are many rolling mills that can be provided with the rolls of these dimensions, e.g., ULS100 (Šmeral, Brno, Czech Republic), QKW1000 (Lasco, Coburg, Germany), H1000 (Beijing University of Science and Technology, China), and D46-100 × 800 (Beijing Research Institute of Mechanics and Electrical Technology, China) [21].

**Figure 11:**
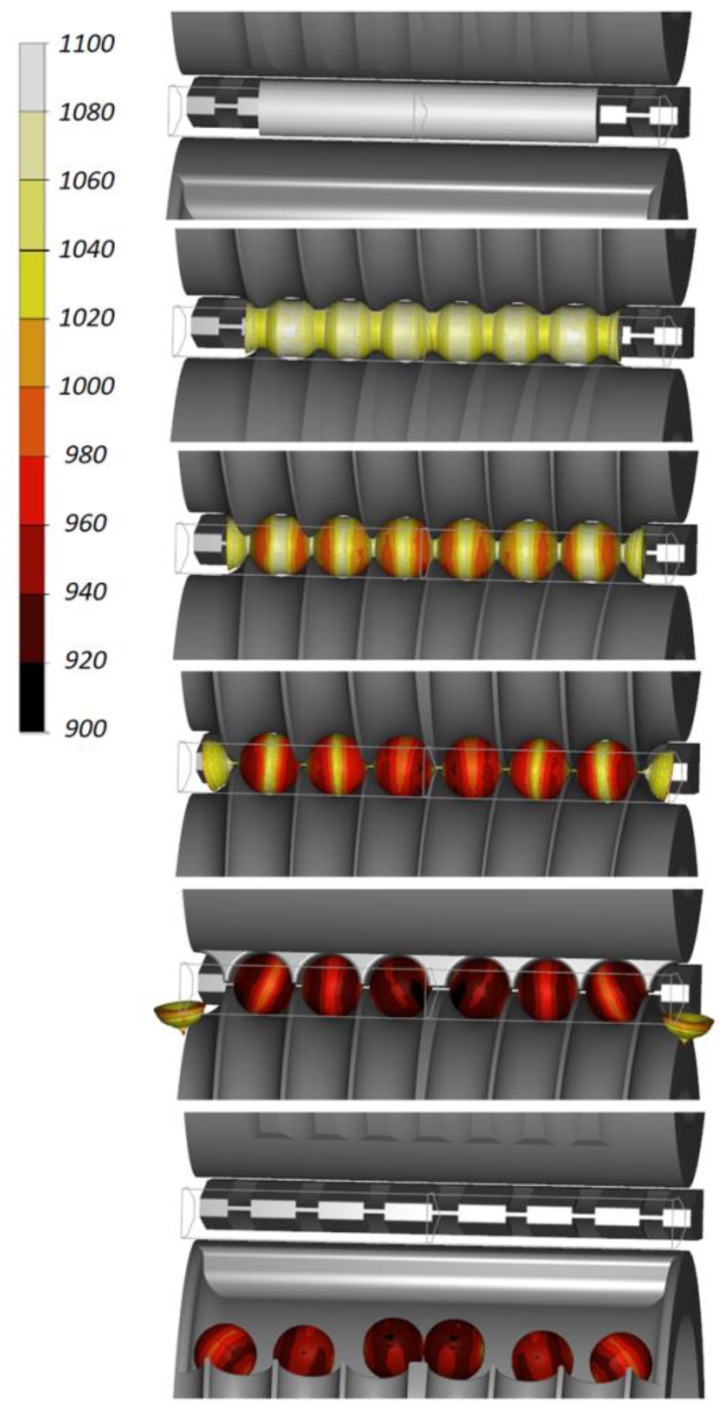
Workpiece shape progression during the cross rolling process for producing six balls with a diameter of 100 mm, and the distribution of temperature (in °C).

As in the previous case, the proposed solution was verified by numerical modelling. The forming process is shown in Figure 11. Balls formed by the flanges have the desired shape. After leaving the sizing zone, the balls get into a special pocket located on the lower tool and thereby leave the working space of the machine.

**Figure 12:**
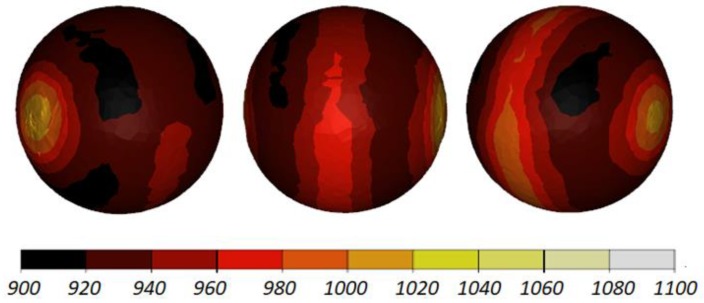
Shape of 100 mm diameter balls produced by cross rolling with two rolls and the distribution of temperature (in °C); the balls are shown in the order from ball in the middle (**left**) to ball at the side (**right**).

The shape of the balls produced with this method is nearly ideal (Figure 12). The balls are practically free from any remnants of the connectors, as was the case in the rolling process with the use of flat tools. This seems to be a result of maintaining the accurate position of the workpiece during forming due to the use of the linear guides. In contrast, in the previously discussed rolling method, the workpiece is rolled over the fixed lower tool.

**Figure 13:**
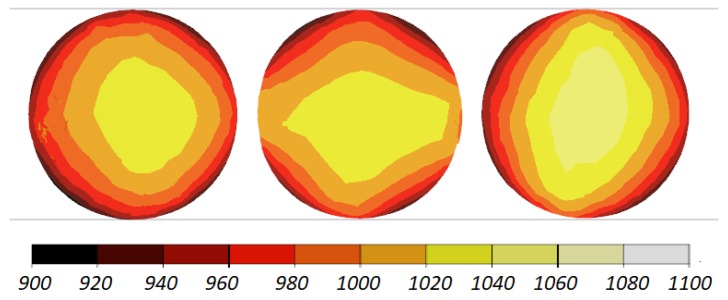
Distribution of the temperature (in °C) inside 100 mm diameter balls produced by cross rolling with two rolls; the balls are shown in the order from ball in the middle (**left**) to ball at the side (**right**).

**Figure 14:**
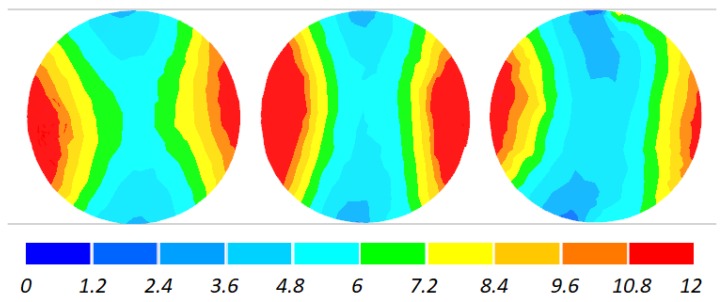
Distribution of effective strains inside 100 mm diameter balls produced by cross rolling with two rolls; the balls are shown in the order from ball in the middle (**left**) to ball at the side (**right**).

The workpiece was maintained in the working space of the machine in the desired position during the rolling process, which is proven by the fact that all balls have similar distributions of the temperature (Figure 13) and effective strains (Figure 14). The temperature of the balls produced with the use of two rolls is slightly higher (approximately by 20 °C) than that of the balls produced by rolling with the flat tools. This probably results from more intensive plastic working due to the higher width of the flanges.

**Figure 15:**
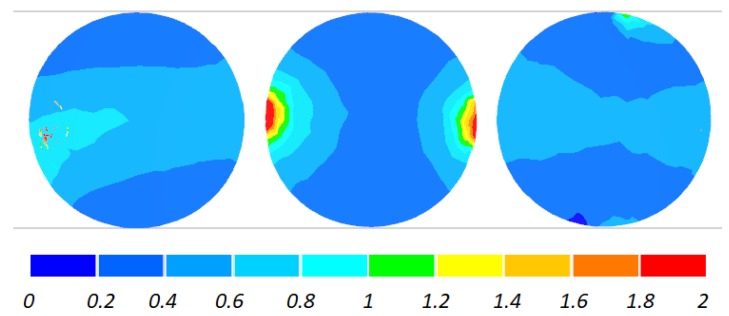
Distribution of the damage function (according to the Cockcrof–Latham ductile damage criterion) inside 100 mm diameter balls produced by cross rolling with two rolls; the balls are shown in the order from ball in the middle (**left**) to ball at the side (**right**).

The two-roll variation of the proposed rolling method does not bring about any significant changes in the distribution of the ductile damage criterion, as shown in Figure 15. In the entire cross section of the ball, the damage criterion is lower than the assumed critical value, which means that balls produced by the rolling method based on the use of two rolls should also be free from internal cracking.

**Figure 16:**
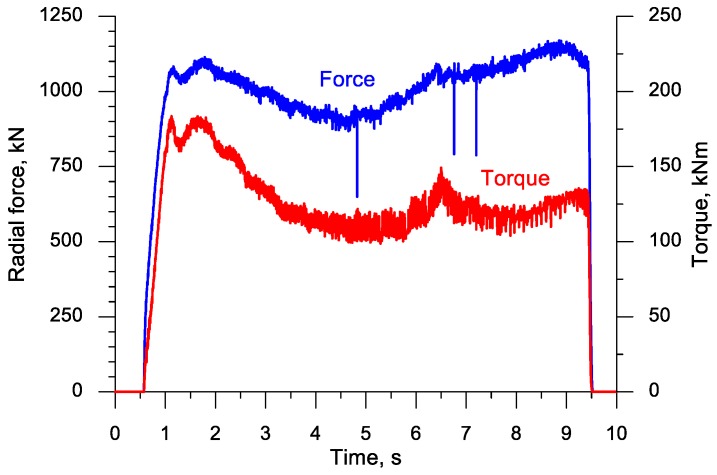
Variations in the force parameters when producing six balls with a diameter of 100 mm by cross rolling with the use of two rolls.

Figure 16 shows the plot of the radial force (acting on the tools) and the torque on one of the rolls. In this rolling case, the radial force does not change as clearly as in the rolling process with the flat tools (the highest forces are similar), which can be explained by the use of higher width flanges and better filling ratio in the forming stage. The torque is highest at the beginning of the forming process, i.e., when the higher width flanges cut into the workpiece.

## 3. Experiment

**Figure 17:**
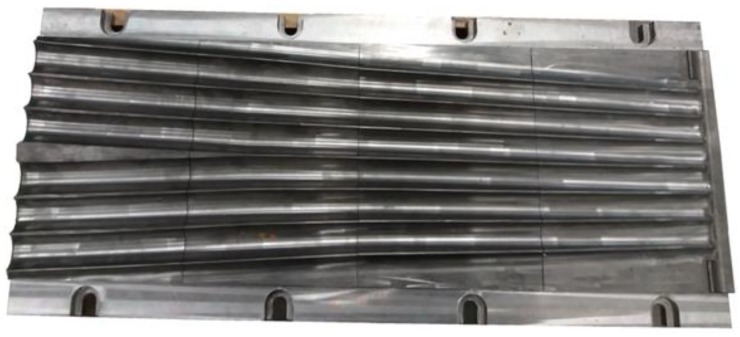
One of the tools used in the experimental cross rolling process for producing six balls with a diameter of 40 mm.

Given the very good numerical results, the proposed rolling method for producing balls was then verified under laboratory conditions at the Lublin University of Technology. Owing to the available equipment, the experiments involved producing six balls of 40 mm in diameter using the flat tools. The tools shown in Figure 2 were resized to 1:2.5 scale. The experimental tools were made of hot-work tool steel heat-treated to have the hardness between 44 and 46 HRC. One of thereby obtained tools is shown in Figure 17. Next, the tools were mounted in a laboratory rolling mill with a hydraulic drive, capable of producing parts with the diameter range between 20 and 70 mm.

**Figure 18:**
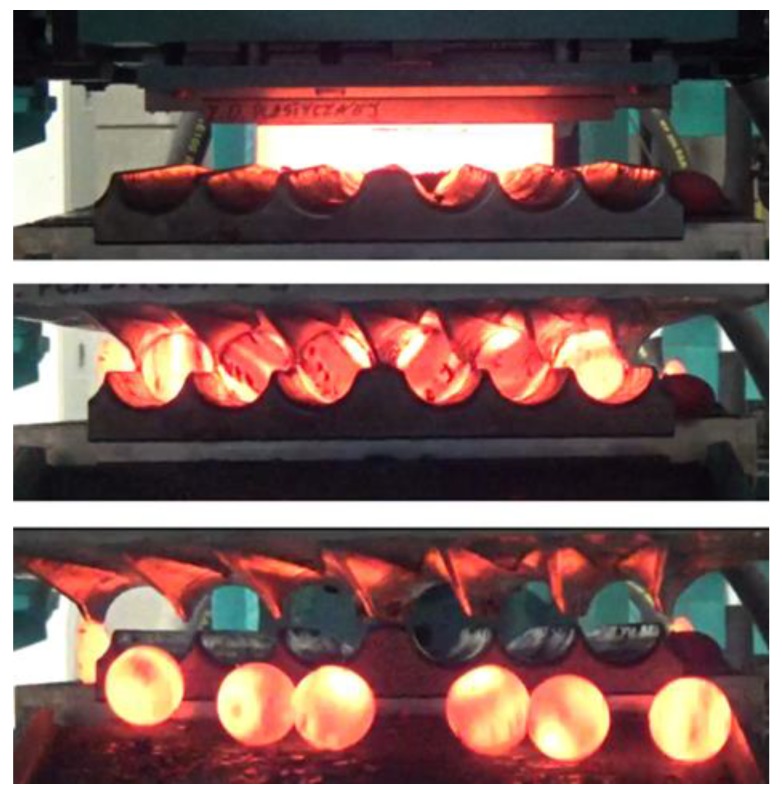
Production of six balls with a diameter of 40 mm by cross rolling with the use of flat tools: billet is positioned on the lower tool (**top**); advanced stage of the rolling process (**centre**); and produced balls leave the machine rolling down into the container (**bottom**).

The experiments were performed using a steel billet of 36 mm in diameter and 208 mm in length, preheated to the temperature of 1100 °C. After preheating, the billet is placed on the lower fixed tool in a specially designed billet-positioning sleeve. The power is switched on and the upper tool moving at the speed of 0.3 m/s rotates the workpiece over the lower tool. Balls formed due to the action of the flanges roll out from the roll pass to the container. One of the performed rolling experiments is shown in Figure 18.

**Figure 19:**
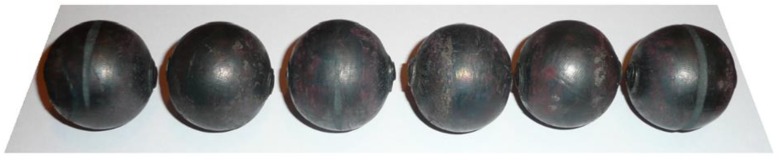
Steel balls of 40 mm in diameter produced by cross rolling, obtained from the experimental tests.

**Figure 20:**
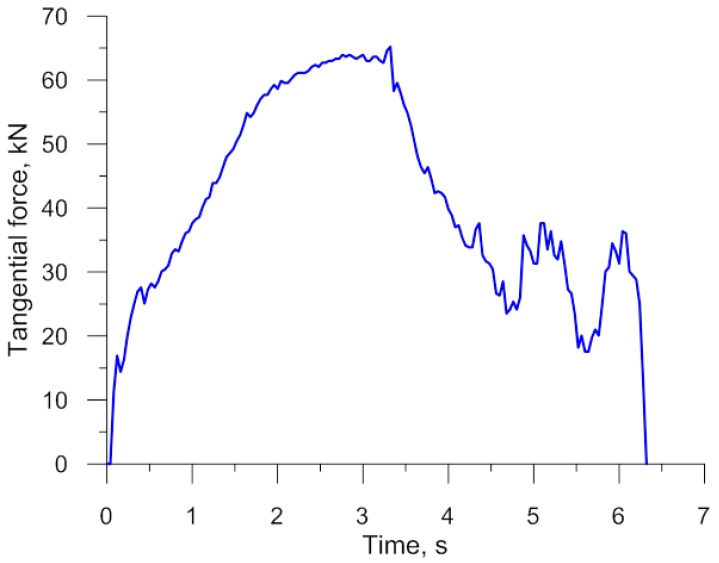
Variations in the tangential force in the rolling process for producing six balls with a diameter of 40 mm.

A set of six simultaneously produced balls is shown in Figure 19. Their shape is correct and they have a diameter of $Ø40.2_{-0.5}^{+1.1}$ mm. On the circumference of the side balls one can see very small underfill, which results from the fact that a certain amount of material is transferred to the end scraps. In the points where the balls were separated, one can notice small remnants of the material, the shape of which is identical to that obtained in the numerical simulation (Figure 4). It should however be mentioned that both the shape and size of the produced balls meet all requirements for balls used as grinding media. The experiments also involved the measurement of the radial force responsible for the motion of the moving tool. The behaviour of this force, plotted in Figure 20, shows a qualitative agreement with the numerically modelled case of rolling 100 mm diameter balls (Figure 8). The only differences can be observed during the ball sizing stage, as the real process is characterized by slightly higher variations in the radial force, which is probably caused by the removal of the remainders of the connectors between adjacent balls. The plots do not agree in quantitative terms. This results from the fact that Figure 8 shows the tangential force in rolling 100 mm diameter balls, while the plot in Figure 20 illustrates variations in the tangential force during the rolling of balls with a diameter of 40 mm. Given the tool dimensions constraints (size of the rolling mill), the experimental verification of the rolling process for producing 100 mm diameter balls was carried out in a 1:2.5 scale by rolling balls with a diameter of 40 mm.

## 4. Conclusions

The numerical modelling and experimental tests lead to the following conclusions:

- The proposed cross rolling method can be used to form six balls at the same time, each ball with a diameter of 100 mm. When rolling six balls with a diameter of 100 mm, the minimal length of the rolls should be equal to 800 mm. If the rolls have a smaller width, the number of simultaneously produced balls should be decreased.
- The proposed cross rolling process for producing balls can be performed either with two flat tools or with the use of two rolls. Rolling mills equipped with two rolls are more widely available on the market. When the rolling process is performed with the use of rolls, the rolling mill should also be equipped with two guides to maintain the correct position of the billet in the working space of the machine.
- The accuracy of produced balls is not very high. The shape and dimensions of balls produced by the proposed method meet the requirements for balls used as grinding media in ball mills. The dimensional tolerance of balls for grinding media is ±3 mm.
- Balls produced by cross rolling with two rolls have higher shape and size accuracy, as the workpiece is precisely positioned in the working space of the rolling mill. An incorrect position of the billet can lead to various defects, such as underfill of one of the side balls, overfill due to a skew position of the billet, and the occurrence of cracks in the centre line of the balls.
- The proposed rolling method for producing 100 mm diameter balls is characterized by high throughput, amounting to 7.2 t/h for the two-roll variation of the process. The use of rolling mills equipped with two rolls prevents idle running of the tools that would lead to a lower efficiency of the rolling process. The throughput of the rolling process also largely depends on the efficiency of billet heating.
- The temperature of produced balls is high enough to perform quenching without material reheating. If the temperature of produced balls is too high, the balls should be subjected to slow cooling on the conveyors transporting them to the quenching tank. On the other hand, the temperature cannot be too low, as this will prevent a correct realization of the quenching process.
- The proposed cross rolling process for producing 100 mm diameter balls can be realized using commercial rolling mills available on the market. As a result, the cost of implementing the new manufacturing technique will be reduced. Moreover, rolling mills of this type can be used for rolling other axisymmetric parts, which will contribute to higher manufacturing flexibility of a production company.
